# Ocular Phantom-Based Feasibility Study of an Early Diagnosis Device for Glaucoma

**DOI:** 10.3390/s21020579

**Published:** 2021-01-15

**Authors:** Marie-Valérie Moreno, Cloé Houriet, Pierre-Alain Grounauer

**Affiliations:** 1 Research Center, RunSys, 53 Avenue Carnot, 69250 Neuville-sur-Saône, France; 2 Research Center, Fabrinal, Rue de la Tuilerie 42, 2300 La Chaux-de-Fonds, Switzerland; cl.houriet@fabrinal.ch (C.H.); grounauer@visiocard.ch (P.-A.G.)

**Keywords:** eye impedance, ocular phantom, glaucoma

## Abstract

Glaucoma causes total or partial loss of vision in 10% of people over the age of 70, increasing their fragility and isolation. It is characterised by the destruction of the optic nerve fibres, which may result from excessively high intraocular pressure as well as other phenomena. Diagnosis is currently reached through a combination of several checks, mainly of the eyes’ fundus, tonometry and gonioscopy. Prior to validation for human subjects, the objective of this study is to validate whether ocular phantom-based models could be used to diagnose glaucoma using an onboard system, which could, even at home, prevent the early-stage development of the pathology. Eight phantoms modelling healthy eyes and eight phantoms modelling eyes with glaucoma due to excessive intraocular pressure were measured using an onboard system, including lens and electrophysiology electronics. We measured the actual average Zr (real part of impedance) impedance of 160.9 ± 24.3 ohms (glaucoma ocular phantom models) versus 211.9 ± 36.9 ohms (healthy ocular phantom models), and an average total water volume (Vt) of 3.02 ± 0.35 mL (glaucoma ocular phantom models) versus 2.45 ± 0.28 mL (healthy ocular Phantoms). On average, we obtained 51 ohms (−24.1%) less and 0.57 mL (22.9%) of total water volume more, respectively. Normality tests (Shapiro–Wilk) for Vt and Zr indicate *p* < 0.001 and *p* < 0.01, respectively. Since these variables do not respect normal laws, unmatched Mann–Whitney tests were performed indicating a significant difference between Vt and Zr in the healthy ocular phantom models and those modelling glaucoma. To conclude, this preliminary study indicates the possibility of discriminating between healthy eyes with those with glaucoma. However, further large-scale studies involving healthy eyes and those suffering from glaucoma are necessary to generate viable models.

## 1. Introduction

Glaucoma causes partial or complete loss of vision in 10% of people over the age of 70, increasing their fragility and isolation. It is characterised by destruction of the optical nerve and retinal cells. In some cases, an increase in intraocular pressure is the one of the first sign of the illness. In other cases, the pressure is normal and nothing makes it possible to prevent the development of the illness. Diagnosis is currently reached through several related checks on the eyes’ fundus, tonometry, gonioscopy, visual fields, optical coherence tomography (OCT) and flicker ERG (Electro Retino Graphy), for instance. In fact, it is still difficult to diagnose this pathology. We hypothesise that this loss is correlated with a decrease in ocular electrical impedance due to an increase in water percentage.

An electric current applied to the eyes facilitates the entry of molecules and has been used for therapeutic purposes [1]. Some studies have been conducted to electrically stimulate the retina through a contact lens equipped with an emitting electrode, such as the ERG-jet sensor [2]. The authors have injected a current of up to 5 mA.

Some preliminary studies have shown that bioimpedance (an analytical measurement of living beings’ electrical characteristics that is low cost, fast and easy to use) may be of interest for exploring various tissues, depending on the pathology, as it is a non-invasive, rapid and inexpensive method. To do so, this technology requires the injection of a low current in the range of 8 to 800 µA.

Fukuda et al. [3] designed a study to determine the validity of living electrical corneal resistance (CR) on 50 subjects. They demonstrated a significant difference between healthy and injured eyes.

Jürgens et al. [1] performed multi-frequency impedance measurements (from 10kHz to 10 MHz) on pigs’ eyes. They demonstrated they could analyse the electrical parameters of aqueous and vitreous humours such as the crystalline lens (nucleus and cortex).

**Figure 1:**
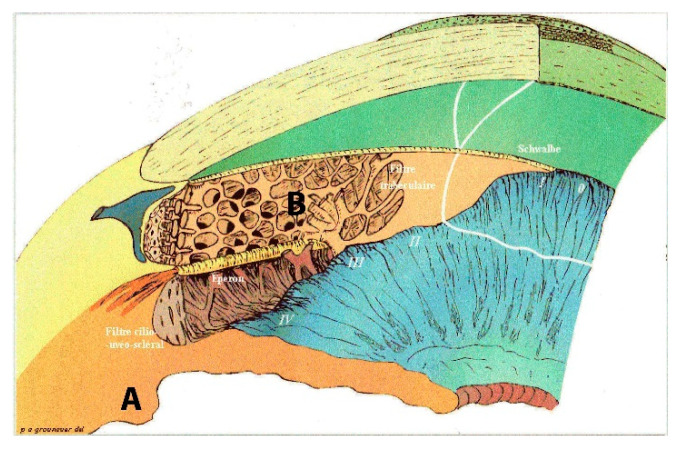
Goniogram 3D inner eye aqueous circuitry. **A** ciliary process production. **B** trabecular meshwork resorption impaired in hypertensive glaucoma patients and normal in normal intraocular pressure (IOP) glaucoma patients. 0 to IV: filtration corneoscleral angle degrees.

Several eye diseases involve an increase in intraocular pressure (IOP), including glaucoma, uveitis and diabetic retinopathies. Ageing also modifies those values [4,5,6]. As a reminder, aqueous humour is produced in the posterior chamber of the eye and is expelled after being filtered through a kind of sponge called the trabecular meshwork (Figure 1). There is an equilibrium between production and resorption that maintains the pressure between 15 ± 2 mm Hg. If the aqueous humour encounters resistance to evacuation, it accumulates in the anterior chamber. With age, filtration of the aqueous humour may be more difficult and cause an increase in IOP, which is not always harmful to the eye [7].

Moreno et al. [8] have shown interest in using a specific lens associated with a bioimpedance analyser to evaluate the electrical properties of the aqueous humour that appears to correlate with the aging of the eye.

Glaucoma with normal intraocular pressure is still not a well understood disease. We suppose that it arises from compression of the optic nerve at the back of the eye, on the retina. Glaucoma with high intraocular pressure comes from a dysfunction between secretion and resorption balance in the trabeculum. Figure 1 shows (A) the ciliary process behind the iris where aqueous humour is produced, and (B) the trabecular meshwork in the angle formed by the iris and posterior corneal surface localisation of the resorption canalisation system.

In normal pressure glaucoma, the aetiology is unclear and many reasons can be assumed, from genetic abnormality to metabolic dysfunction and other pathologies described by Killer et al. [9], Esporcatte et al. [10] and Berdahl et al. [11].

This type of water and filtration problem has been widely studied, notably using bio-impedance technologies [12]. Those technologies examine the electrical properties of tissues under the influence of currents of variable frequencies. Tissues contain fluids and ions, and thus, resistivity and then resistance R (ohm) can be associated with them, according to the following elements.

The resistivity of an aqueous solution (sol) ρ is defined according to Equation (1):

ρ_sol_ = 1/σ_sol_ (1)

where

(2) $$\sigma_{sol}=F\cdot \Sigma \left(\left|z_{i}\right|\cdot c_{i}\cdot u_{i}\right)\sigma_{sol}=F\times \Sigma \left(\left|\varepsilon_{i}\right|\times C_{i}\times u_{i}\right)$$

and

(3) $$\sigma_{i}=\left|z_{i}\right|\cdot c_{i}\cdot u_{i}\cdot F\sigma_{i}=\left|\varepsilon_{i}\right|\times C_{i}\times u_{i}\times F$$

where |*z_i_*| is the absolute value of the charging of an ion;

*C_i_* is the concentration of this ion (i) in mol/m^3^;
*U_i_* is the ion (i) mobility in m²/s.V;
*F* is 1 faraday or 96,500 C/mole;
*σ* is conductivity in S.m^−1^ and ρ is resistivity in Ω.m.

The resistance of extracellular or intracellular fluids is then obtained according to:

R = ρ.l/S (4)

where

R is resistance (Ω);
l is the height of the schematic column containing the solution (m);
S is the surface of the schematic column containing the solution (m²);
ρ is the resistivity of the liquid inside the column (Ω.m).

**Figure 2:**
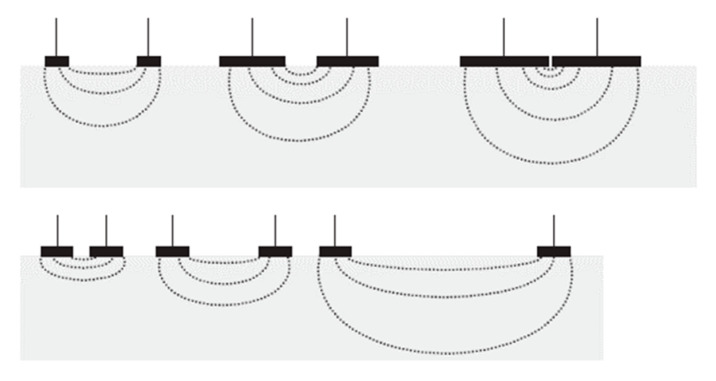
Sensitivity in deep layers according to the distance and the dimensions of the electrodes [10].

To measure these resistances, electrodes are applied to the patients. Grimnes et al. [13] explain the relationship between the sensitivity of the measure according to the depth of tissue being studied and the increase in distance between the electrodes and/or the increase in surface area of the electrodes (Figure 2).

It is in this context that we placed the electrodes in the lens to access the anterior chamber of the eye.

According to Kanai and Meijer [14,15], the cell membrane behaves like a capacitor. Indeed, a membrane is made up of phospholipids and is therefore insulating. When current encounters a cell membrane, electrical charges build up on both sides of the membrane without being able to pass through it. An electric double layer is then created, which forms a capacitor. A reactance *X* (ohm) can be associated to this phenomenon (Equation (5)). These data vary according to the frequency.

(5) $$X=\frac{1}{jCm\left(2\pi f\right)}$$

where *X* is the reactance (Ω), *f* the frequency of the injected current in Hz and *Cm* the capacity of the membrane in Farad.

In that model, the extracellular resistance is *Re* (obtained to a theoretical f = 0 Hz) and the total resistance of the tissue is *R*∞ or *R_inf_* (obtained to a theoretical infinite frequency).

The impedance (the sum of the resistive and capacitive properties) of the tissue measured is then a complex number which can be graphed in a complex plan (the imaginary part of the impedance Zimg versus the real part Zr).

**Figure 3:**
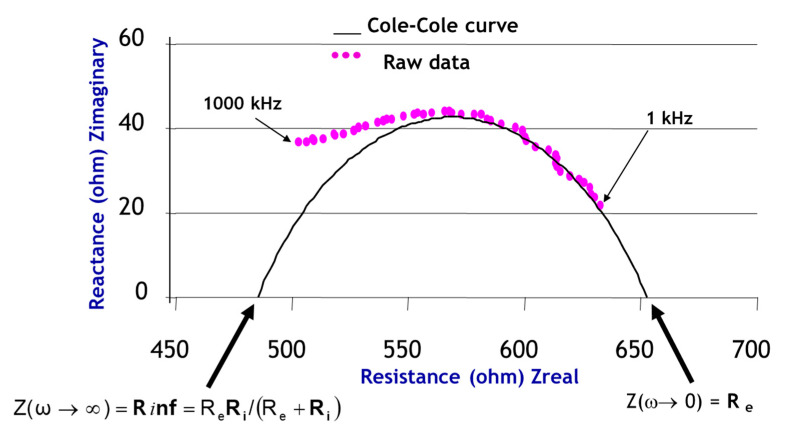
Cole-Cole curve on raw data in the impedance complex plan (ω = 2π*f* in rad.s).

As described by Bonnet et al. [16], we can fit a Cole–Cole curve (Figure 3) to the raw data to obtain new parameters. Unlike the authors, we do not use the Kasa method, but the nonlinear least squares method. Those parameters characterise biological tissues. We use them as *Rc*, *Xc*, *Fc*, *τ*, alpha, *Cm, Re*, *R*_∞_ to model electrical data and characterise the eyes in the study.

Other parameters using the same model enhance the equations:

- *C* (xc, yc) (centre of the Cole–Cole circle, r: radius of the Cole–Cole circle);
- *Rc*, *Xc* and *Fc* characteristic data of the curve bend (characteristic resistance, characteristic reactance and characteristic frequency);
- *Ri* (modelled resistance of the intracellular area in ohms);
  (6) $$R_{i}=\frac{R_{e}R_{\infty }}{R_{e}-R_{\infty }}R_{i}=\frac{R_{e}R_{\infty }}{R_{e}-R_{\infty }}$$
- Alpha α (phase angle in degrees at the characteristic frequency);
  (7) $$\alpha =atan\left(\frac{Xc}{Rc}\right)\cdot \frac{180}{pi}=atan\left(\frac{yc+r}{xc}\right)\cdot \frac{180}{pi}\alpha =atan\left(\frac{Xc}{Rc}\right)*\frac{180}{pi}=atan\left(\frac{yc+r}{xc}\right)\cdot \frac{180}{pi}$$
- Tau *τ* (ionic relaxation time in µs);
  (8) τ=(Re+Ri)∗Cmτ=(Re+Ri)∗Cm
  where *Cm* is the cell membrane’s capacitance in Farad, and therefore,
  (9) $$Cm=\frac{1}{2\pi Xc}=\frac{1}{2\pi \left(yc+r\right)}Cm=\frac{1}{2\pi Xc}=\frac{1}{2\pi \left(yc+r\right)}$$

Prior to a validation on human subjects, the objective of this preliminary study is to validate on phantoms whether a glaucoma diagnosis could be reached using an onboard electrophysiological system, which could, even at home, prevent the development of the pathology at an early stage.

## 2. Materials and Methods

### 2.1. Phantoms

The animal model, although relevant, remains cumbersome to implement, and certain electrical characteristics of small mammals, such as rodents, remain far from human characteristics, including metabolic differences (the average heart rate of a rodent can be up to 250 to 450 beats per minute [17]).

Phantoms are also limited in their representativeness of human tissue but are easy to implement [18]. Among major differences between hydrogel eye simulations and in vivo eyes are that liquids in the latter permanently renew, changing and moving, e.g., red blood cells and temperature ratio between external/internal cornea [19].

Several studies have described the development of techniques and the ingredients for preparing materials that mimic human tissue [20]. The most commonly used materials are based on water, agarose, gelatine and gels. Agarose and gelatine-based phantoms (also known as hydrogels) are the most widely-used alternatives. Agarose is used to obtain consistency, NaCl to increase conductivity and fine graphite to simulate conductance in phantoms.

Although these phantoms are stable for several weeks [21], microbial developments may occur, causing measurement errors. Moreover, a precise preparation procedure must be followed to optimise the reliability of the measurements: cooking time, order and mode of integration of ingredients, cooling time, resting time at room temperature before measurements, etc. [22,23].

Jurgens et al. [1] determined the eye’s resistive properties, described in Table 1.

A healthy eye has an average intraocular (anterior chamber) pressure of 15 mmHg, while an eye with abnormal IOP (e.g., glaucoma) has an overpressure of 21 mmHg, which is about 30% higher. According to Tavernier et al. [24], there is a negative linear relationship between pressure and resistivity variations. Either the resistivity of eyes with glaucoma will present 30% *ρ_glaucoma_* less than that of a healthy eye, or about *ρ_glaucoma_* = 0.465 Ω.m.

Applying Equations (1)–(3), we obtained the corresponding mole number of NaCl needed to be added to the solution to obtain the expected resistivity.

According to Equation (9), the amount of salt to be added to the phantoms representing eyes damaged with glaucoma is determined by:

(10) $$n=\frac{m}{M}$$

with

*n:* number of moles in mole;
*m*: sample mass in g;
*M*: molar mass in g.mol^−1^ (i.e., 58.5 g.mol^−1^ for NaCl).

**Figure 4:**
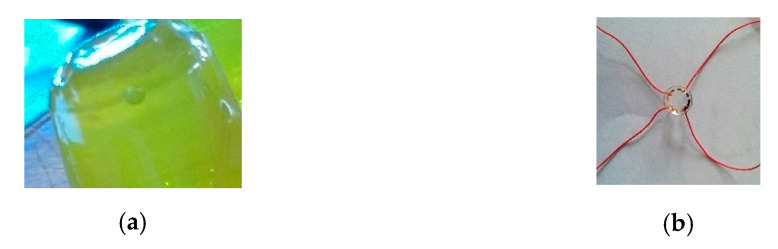
Picture of one of the phantoms representing a healthy eye (**a**) and the associated lens (**b**).

The phantoms present themselves in cones with 2.5 cm diameter round ends. Table 2 presents the composition of the different phantom types:

Figure 4 shows an example of a phantom representing a healthy eye and an example of an associated lens. The top of the phantom, oval in shape, simulates the cornea and the anterior chamber of the eye. The lens, fitted with its four electrodes, is then placed in contact with this part of the phantom as for a real eye. The four wires of the lens are connected to a bioimpedance board.

Experimental conditions (temperature, baking and moulding) have made it possible to preserve eight healthy eye phantoms (S1 to S8) and eight eye phantoms presenting glaucoma (G1 to G8). These populations were randomly separated into two subpopulations, one for model creation and the other for validation.

Five prototype lenses were tested to study the variability between the electrodes.

The measurements for each lens were made randomly on the S or G phantoms.

### 2.2. Materials

**Figure 5:**
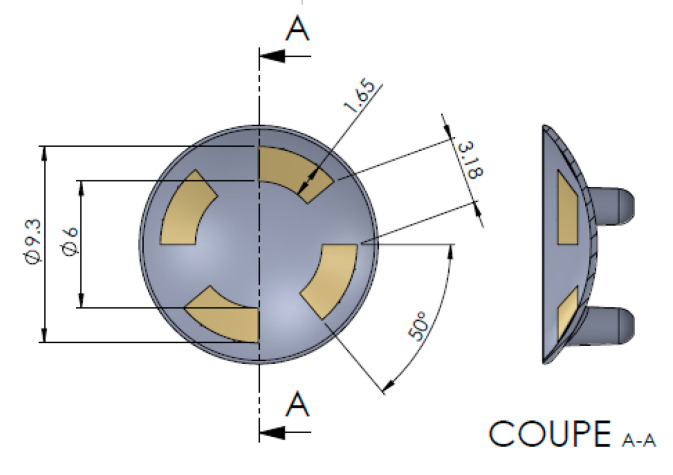
Diagram of an Impedance Eye Recording (IER) Lens injected in Styrene-Methyl Methacrylate (SMMA) with thin gold layer electrodes.

Figure 5 shows the schema of the lenses used. Measurements were taken using the Focus Impedance Measurement method [25] which improved zone localisation compared to conventional methods, particularly adapted to our need of focusing on the anterior chamber. These lenses (Fabrinal, Switzerland) were connected to an electrophysiological module (ϕ-module, RunSys, France; mean accuracy on test bench (from 1 to 300 kHz) respectively on *R* and *X*, 1.02 ± 1.18% and 1.18 ± 1.45%, mean repeatability on test bench 0.0157 ± 0.0039 and 0.0425 ± 0.0227%; bypass HPF (high-pass) and LPF (low-pass) filters, amplifier 10 V/V, sampling rate 64 sps) to form the Impedance Eye Recording (IER). A current of 32 µA, according to a six-frequency sweep, was injected into phantoms. The Cole–Cole parameters were collected, as well as Zr (real part of the impedance), and Vt (Total water volume in ml) [26]. Then, using an algorithm based on all calculated parameters [27], the device estimated a glaucoma prediction score.

In the context of this feasibility study, only one function of the lens is used (anterior chamber measurement for the observation of an abnormal IOP). In a forthcoming study, the second IER function will be used to explore the posterior chamber of the eye and the area of the optic nerve for the diagnosis of normal IOP glaucoma.

### 2.3. Final Prototype Description

**Figure 6:**
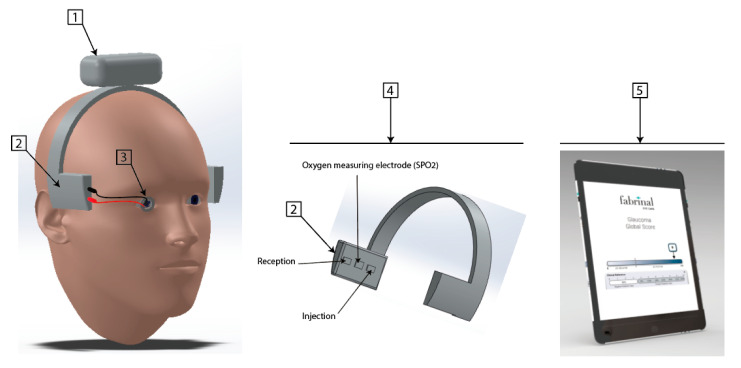
First 3D rendering Impedance Eye Recording (IER).

**Figure 7:**
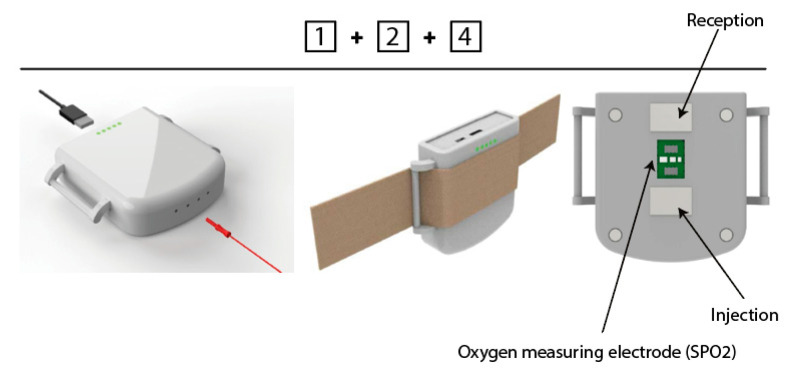
Evoluted 3D rendering Impedance Eye Recording (IER).

The IER prototype designed for the clinical study (Figure 6 and Figure 7), which is still under development, is based on four parts:

1. An electronic part composed of a battery, Bluetooth Low Energy;
2. An electronic part composed of two types of sensors: a PPG (Photo Plethysmo Graph) sensor for SpO2 (oxygen saturation of the blood microcirculation of the ophthalmic artery based on optical analysis, based on three LEDs (536, 660 and 940 nm) and two photodiodes, sample rate 100 Hz, pulse width 115.2 ms) and a bioimpedance sensor analysing both chambers of the eye (anterior and posterior);
3. An electrode (lens) allowing the bioimpedance of the anterior chamber when used with the bioimpedance sensors in part 2;
4. A system allowing the temporal electrodes to be positioned;
5. A user interface, under development, to be finalised with the clinical data.

In this study, our aim is to first explore the electrical parameters on phantoms that are unable to represent the complexity of the human eye. We then simplify the device for this purpose, using only the electronic board linked to the lens (Figure 4b).

### 2.4. The Algorithms

Using Equations (10)–(13), the device estimated a glaucoma risk score (developed on a subpopulation of the sample and validated on the remaining subpopulation).

We used a Logit model, also known as logistic regression. More simply, we created this score by a binomial regression. The binary variable to be explained is “glaucoma/healthy” and the explanatory variables are the data measured in the study, more particularly those of bioimpedance.

The glaucoma risk score is estimated by a binomial law, calculated as Equation (10):

(11) Glaucoma Risk Score=1(1+e(−(a+bx1+cx2+…+ixi)))

where *a*, *b*, …, *i* are constants and *x*1, *x*2, …, *xi* are experimental variables of the equation.

In this study, having fewer experimental variables, we used the simplified logistic binomial law (two parameters µ, s), with its density function calculated as Equation (11):

(12) f(x;μ, s)=e−((x−μ)/s)s(1+e−(x−μs))2

Then, its distribution function is calculated as Equation (12):

(13) F(x;μ, s)=11+e−(x−μs)

We note in our study the mathematical “expectation” *E(X*) = μ = 0 and the variance Var (*X*) = s^2^π^2^/3, inducing s = 1.

Then, the obtained distribution function of the tumour risk score is calculated as Equation (14):

(14) F(x)=11+e−(x)

with *x* corresponding to the electrical characteristics measured.

We simulate the total eye water using a derived equation from Jaffrin et al. [26].

## 3. Results

### 3.1. Repeatability of the Phantom Measuring Chain

On the phantom, the measuring chain generates an average variation coefficient of 3.1 ± 0.8%.

### 3.2. Inter-Lens Variability

The “lens” factor, according to the Shapiro–Wilk test, indicates *F* = 2.852 with *p* > 0.05. The inter-electrode variability is not significant.

### 3.3. Raw Data

Table 3 shows the raw data obtained for each phantom.

**Figure 8:**
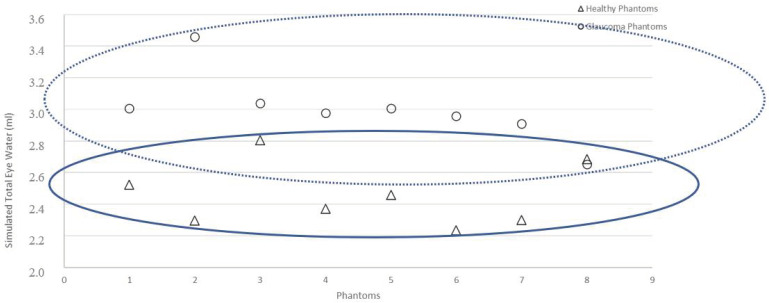
Graphs representing Vt (ml) for each phantom: healthy and simulated glaucoma

Figure 8 shows two groups of data according to healthy or glaucoma phantoms.

We observe that three phantoms, G8, S8 and S3 could be interpreted as “healthy” or “glaucoma”.

### 3.4. Detection of the Presence of Glaucoma by the System

We observed (Table 4) an average real impedance Zr of 160.9 ± 24.3 ohms (glaucoma phantoms) versus 211.9 ± 36.9 ohms (healthy phantoms), as well as an average total water volume of 3.02 ± 0.35 mL (glaucoma phantoms) versus 2.45 ± 0.28mL (healthy phantoms); that is to say, an average difference of 51 ohms (−24.1%) and 0.57 mL (22.9%) respectively. Normality tests (Shapiro–Wilk) indicated for Vt and Zr, *p* < 0.001 and *p* < 0.01, respectively. Since these variables do not respect normal laws, unmatched Mann–Whitney tests were performed, indicating a significant difference between the Vt and Zr of healthy phantoms and those modelling glaucoma.

We observed (Table 5) a risk score higher than 78.8% for phantoms modelling glaucoma, compared to a lower than 26.5% score for healthy phantoms.

## 4. Discussion

From a medical point of view, the first step, with phantoms eyes, allows in vitro recordings to avoid in vivo testing, which is more complicated to carry out. This relatively new method is pertinent and used by many researchers. It has the advantage of proving the feasibility, mathematical model and methodological flaws without having to study subjects. In the particular case of glaucoma, it has been confirmed that this pathology is consequently due to a loss of retinal ganglion cells.

Relative repeatability of the measurement chain (presumably due to the humidity of the phantom surface) has probably impacted the discrimination level of the system, with three phantoms that can be graphically interpreted as either healthy or glaucoma. For S phantoms, we can guess that the too-low impedance, close to those of G phantoms, comes from the thin layer of water that appeared during the experiment due to the high temperature and difficulty of control in the experimental room. Conversely, for the G phantom whose too-high value is close to that of the S phantoms, one can assume a bad positioning of the lens, generating an imperfect contact. It is noted that it was not possible to carry out these experiments in a temperature-controlled environment. Although the phantoms were kept in a refrigerator, the setting time at room temperature (with an outside temperature of 34 °C) generated remarked humidity in the measurement area, which may explain this variation coefficient.

Although they were prototypes, the five lenses tested did not show significant variability between them. We can suppose that the process to deposit gold in the lens according to the quadripolar pattern is reliable.

Although this study is on phantoms, we can derive from this that the risk score obtained is not 0% (for healthy phantoms) and 100% (for glaucoma phantoms). It was also noticed that a strict procedure is needed to ensure a satisfactory repeatability level (eyelid movement control, eye movement control, etc.). Our estimate of the total eye water, although consistent with those obtained for the G versus S phantoms, overestimates the water volumes by a factor of 10. This should be corrected and calibrated on human eyes.

In addition, a dedicated compensation will be developed for the final measurement chain to optimise its accuracy.

The method tested in this study has the drawback of not analysing types of glaucoma at normal intraocular pressure, which are the most difficult to diagnose. This will be done with the complete system (in particular, including the oxygen saturation quantification of the ophthalmic artery) in a clinical study on human subjects. In addition, age disorders and various optical disorders could not be simulated equally.

The results obtained require refinement due of the simplicity of the phantoms compared to a real eye. The different types of tissues and cells and the ionic and hydric transfers between them will impact the robustness of the models. However, new models will be established including, notably, the capacitive effects of tissues, among other things. It is expected that these reactance parameters, on a real eye, will provide us with information on the permeability of the trabeculum in particular.

More generally, a larger, human population will be needed to confirm or refute these preliminary results.

## 5. Conclusions

This feasibility study seems to indicate that the device was able to discriminate between phantoms modelling glaucoma with abnormal intraocular pressure and healthy phantoms in a significant way using electrical measurements. It will be necessary to validate the system on human subjects. To increase the sensitivity and specificity of the system for the global pathology (normal and abnormal IOP), the capacitive characteristics of the tissues, as well as the vascular characteristics of the ophthalmic artery, will be required to enrich human models.

Knowing in addition that diabetic retinopathy is accompanied by alterations of the retinal capillary walls, allowing visible water leakage under the aspect of oedema, we hypothesise that the device could even become a predictive test on that other pathology. Other ailments are also accompanied by capillary alterations and extracellular fluid accumulations, for example: intraocular inflammation or uveitis, various forms of age-related macular degeneration (AMD) and certain intraocular tumours. We plan to explore them using the complete system.

## 6. Patents

The Patent FR2005831 results from a part of this work.

## Figures and Tables

**Table 1:** Characteristics of the resistivities of the different components of the eye [1].

	ρHealthy (Ω.m)
Saline Solution (NaCl 9g.l^−1^)	0.625
Aqueous Humour	0.60–0.67
Glass Humour	0.63–0.66
Cornea	0.39–0.48

**Table 2:** Definition of phantom characteristics.

	Healthy Eye (Yellow Eye)	Glaucomatous Eye (Red Eye)
Composition	4 g/L Agarose 1l Demineralised Water 9 g/L NaCl	4 g/L Agarose 1l Demineralised Water 10 g/L NaCl

**Table 3:** Raw data (means of various lens) obtained for each phantom.

**“Glaucoma Phantom”**	**G1**	**G2**	**G3**	**G4**	**G5**	**G6**	**G7**	**G8**
Zr (ohm)	149.51	177.70	147.58	162.25	150.09	158.42	162.58	181.54
Total Eye Water (mL)	3.01	3.46	3.04	2.98	3.00	2.96	2.91	2.65
**“Healthy Phantom”**	**S1**	**S2**	**S3**	**S4**	**S5**	**S6**	**S7**	**S8**
Zr (ohm)	196.53	223.31	169.16	222.63	217.71	245.11	230.84	183.20
Total Eye Water (mL)	2.52	2.30	2.81	2.37	2.46	2.24	2.30	2.68

**Table 4:** Description of data obtained on Healthy and Glaucoma Phantoms.

	Healthy Phantoms	Glaucoma Phantoms	Mann–Whitney Tests
Average Zr ± SD (Ohms)	211.9 ± 36.9	160.9 ± 24.3	*p* < 0.01
Average Difference ± SD (Ohms, %)		51.0 − 24.1% (**)	
Average Vt ± SD (ml)	2.45 ± 0.28	3.02 ± 0.35	*p* < 0.001
Average Difference ± SD (ml, %)		0.57 − 22.9 (***)	

Real impedance (Zr, Ohms) and total water volume Vt (ml), measured on modelling glaucoma phantom versus healthy phantoms (**, *p* < 0.01; ***, *p* < 0.001).

**Table 5:** Glaucoma risk score obtained for the validated subpopulation.

Phantom	Glaucoma Risk Score %
G1	78.8
G2	83.4
G7	96
G8	100
S2	26.5
S3	0
S7	0

## Data Availability

Data is contained within the article.
